# Depiction of Sexual Harassment in Medical Television Shows

**DOI:** 10.7759/cureus.11842

**Published:** 2020-12-02

**Authors:** Shayann Ramedani, Sayeh Bozorghadad, Robert P Olympia

**Affiliations:** 1 Medicine, Penn State College of Medicine, Hershey, USA; 2 Emergency Medicine and Pediatrics, Milton S. Hershey Medical Center, Hershey, USA

**Keywords:** medical education, media, television, sexual harassment, sexual misconduct, medical student, resident education

## Abstract

Background and objective

Medical television (TV) shows are known to exaggerate medical scenarios, including relationships among/between medical staff and patients. Unfortunately, sexual harassment occurs within the medical and nursing profession. The objective of this study was to analyze the depiction of sexual harassment in eight popular medical TV shows.

Methods

The first 10 episodes of the first season of eight popular medical TV shows (St. Elsewhere, ER, Scrubs, Private Practice, Grey’s Anatomy, Nurse Jackie, The Good Doctor, and The Resident) were viewed and coded by two reviewers. The data abstracted included demographics of those involved in the sexual harassment and examples of sexual harassment depicted.

Results

The analysis was based on 62 instances of sexual harassment. The victim of sexual harassment was female in 77% (49/62) of instances. The most common relationships depicted pertaining to the acts of sexual harassment were attending physicians toward attending physicians (12/62, 19.3%), interns toward interns (8/62, 12.9%), attending physicians towards interns (7/62, 11.2%), and patients toward attending physicians (5/62, 8.1%). The most common examples of sexual harassment portrayed were telling sexual anecdotes/jokes (23/62, 37.1%), inappropriate touching (12/62, 19.4%), and making sexual comments about appearance, such as body parts or clothes (12/62, 19.4%).

Conclusion

Based on our analysis of medical TV shows, instances of sexual harassment occurred most commonly between attending physicians, and most of them were associated with sexual anecdotes/jokes. Medical and nursing professionals may draw on relevant instances from medical TV shows to discuss how to recognize and deal with sexual harassment in the workplace in order to promote a safe and nurturing environment devoid of harassment.

## Introduction

The emotional and physical bonds we see depicted among characters within television (TV) shows give us a glimpse into both how characters interact with each other and how their environments facilitate these interactions. These interactions occur in different ways, and they are formed either through proximity, similar interests, and physical attraction or through emotional understanding. The interactions, however, are not always as innocent as the plot unfolds. The interactions many characters have within their workplace are often imposing or non-consensual and can go from what is viewed by viewers as “playful,” to harassment. The ways in which the workplace is involved with the romantic interactions of characters on TV are relevant to the perception of that workplace.

One of the places where a considerable amount of TV romance occurs is within the confines of a hospital. This can happen with different members of the clinical team, but when the advances are not welcome, it steers away from the outwardly fun ploy of plot lines and turns into harassment [1]. It has been reported that a third of medical students and residents report being sexually harassed at some point in their clinical training [2]. Cook et al. conducted a self-reported survey study among third-year medical students from 24 different medical schools, which confirmed that 64% of students who responded had experienced at least one incident of mistreatment by faculty [3]. This, in turn, was associated with higher rates of burnout. This is a startling reality that needs to be addressed.

In the current era, when workplace harassment is given proper attention and people are more outspoken about instances of harassment from superiors or colleagues, we have an incredible opportunity to discuss the depiction of such harassment in popular media. Apart from being a relevant use of media to address issues within medicine that are usually uncomfortable to talk about, this can also serve as a method to address the way the field of medicine is perceived by the general public. With the field becoming more diverse, clinicians and clinician-educators want to make the field more accessible and safe for people from different genders, backgrounds, and identities. The impact of watching popular media and dissecting its impact on the way people perceive the field brings two added advantages: the first being that popular medical TV shows are known and/or watched widely and most people have a keen sense of the context regarding what the shows portray. Secondly, we can also change perspectives and appreciate how people view the medical profession and also examine what inherent biases they come in with so that they can be addressed properly. If the general population thinks that seeing someone do something inappropriate and then laughing it off is acceptable in the field of medicine, then it is better to address it through education to cease the perpetuation of that bias.

Medical TV shows are known to depict situations that occur in real-life settings. There have been no published studies analyzing the depiction of sexual harassment in medical TV shows, focusing on the demographics of those involved in sexual harassment and the types of sexual harassment depicted. Through the analysis of eight primetime medical TV shows, we seek to explore the inter-character interactions in them and examine their depiction of sexual harassment.

## Materials and methods

We conducted a content analysis study in 2019 by examining the depiction of sexual harassment in eight popular medical TV shows (Table 1). The shows included St. Elsewhere, ER, Scrubs, Private Practice, Grey’s Anatomy, Nurse Jackie, The Good Doctor, and The Resident. TV shows were chosen based on the time in which they first aired in order to assess if any changes in the frequency of sexual harassment depiction have occurred over time.

**Table 1:** Details of medical TV shows included in our analysis

Medical TV show name	Years aired	Setting	Location
St. Elsewhere	1982-1988	Emergency department	Boston, MA
ER	1994-2009	Emergency department	Chicago, IL
Private Practice	2004-2012	Private practice operating room	Los Angeles, CA
Grey's Anatomy	2005-present	Emergency department and inpatient setting	Seattle, WA
Nurse Jackie	2009-2015	Emergency department	New York, NY
The Resident	2017-present	Emergency department and inpatient setting	Atlanta, GA
The Good Doctor	2017-present	Emergency department and inpatient setting	San Jose, CA

A data collection instrument was developed by the authors in order to record instances of sexual harassment depicted in the first 10 episodes of the first season of each of the eight selected TV shows. Abstracted data for each depicted sexual harassment instance included the demographics of the two characters involved in the sexual harassment scenario (sex, medical role) and the type of sexual harassment (physical or verbal). We determined the relationship of those involved in the sexual harassment instance based on the medical role of the character performing the sexual harassment and the medical role of the victim of the sexual harassment.

According to the U.S. Equal Employment Opportunity Commission, sexual harassment can include unwelcome sexual advances, requests for sexual favors, and other verbal or physical harassment of a sexual nature [4]. Additionally, harassment does not have to be of a sexual nature but can include offensive remarks regarding a person’s sex. For the purpose of this research, instances of sexual harassment or misconduct were categorized based on whether they were physical or verbal in nature. Physical instances of harassment included displaying inappropriate sexual images or posters; staring, whistling, gesturing, or touching oneself in a sexually suggestive manner; and inappropriate touching including pinching, patting, rubbing, or brushing up. Any other instances of physical sexual harassment that did not fit into these categories were further defined by the reviewer. Verbal instances of harassment were defined as requesting sexual favors or dates; telling sexual anecdotes about oneself or others; making sexual comments about another person’s appearance, clothing, or body parts; sharing sexually inappropriate videos or photos; sending suggestive letters, notes, or emails; asking personal questions of sexual nature (i.e., about sexual orientation or sexual history); or making comments about someone’s sexual orientation or gender identity. Other instances of verbal harassment that did not fit into these categories were also further defined by the reviewer.

Two reviewers (S.R. and S.B.) viewed and coded each of the 80 episodes independently. After each episode was independently coded, results were reviewed to identify any discrepancies between the two sets of data until one final data set was attained. Data were imported into Excel spreadsheets, and frequencies for each outcome variable were determined. The Institutional Review Board (IRB) at the Penn State Hershey Medical Center deemed the study exempt from IRB approval.

## Results

A total of 62 instances of sexual harassment depicted in 80 episodes were included in the analysis. The victim of the sexual harassment was female in 77% (49/62) of instances. The most common relationships depicted pertaining to the acts of sexual harassment were attending physicians toward attending physicians (12/62, 19.3%), interns toward interns (8/62, 12.9%), attending physicians towards interns (7/62, 11.2%), and patients toward attending physicians (5/62, 8.1%) (Table 2). The most common types of sexual harassment depicted were telling sexual anecdotes/jokes (23/62, 37.1%), inappropriate touching (12/62, 19.4%), and making sexual comments about appearance, such as body parts or clothes (12/62, 19.4%) (Table 3). Instances of sexual harassment were more frequent in the sample of medical TV shows aired in the 2000s and less frequent in those aired after 2009 (Table 4).

**Table 2:** Relationship of those involved in sexual harassment depicted in our sample of medical TV shows

Relationships	Number of instances (N=62), n (%)
Attending to attending	12 (19.3%)
Intern to intern	8 (12.9%)
Attending to intern	7 (11.2%)
Patient to attending	6 (9.6%)
Patient to intern	4 (6.4%)
Resident to attending	4 (6.4%)
Attending to nurse	4 (6.4%)
Resident to nurse	4 (6.4%)
Patient to nurse	4 (6.4%)
Intern to nurse	2 (3.2%)
Attending to resident	2 (3.2%)
Nurse to intern	2 (3.2%)
Intern to attending	1 (1.6%)
Patient to resident	1 (1.6%)
Resident to resident	1 (1.6%)

**Table 3:** Categories of sexual harassment depicted in our sample of medial TV shows

Type of harassment	Number of instances (N=62), n (%)
Telling sexual anecdotes or jokes	23 (37%)
Inappropriate touching	11 (19%)
Making sexual comments about appearance (body parts or clothes)	11 (19%)
Other (verbal)	6 (9.6%)
Other (physical)	4 (6.4%)
Requesting sexual favors	4 (6.4%)
Displaying inappropriate sexual images	2 (3.2%)
Staring/whistling/gesturing in a sexually suggestive manner	1 (1.6%)

**Table 4:** Total number of instances of harassment and instances per episode stratified by year of the first season

Period	Total number of instances (N=62), n (%)	Total number of episodes	Instances per episode
Pre-2000s	12 (19.3%)	20	0.65
The 2000s	42 (67.8%)	30	1.43
2009-present	8 (12.9%)	30	0.27

## Discussion

The findings of our study suggest that there is a definite theme of sexual harassment that exists across our sample medical TV shows. This trend of sexual harassment within the TV shows itself describes the environment in which the shows are depicted. These instances are happening within the walls of the hospital, in operating rooms, during patient encounters, or even behind clinical team stations. The pervasive ways in which sexual harassment occurs in these shows are indicative of another theme as well: an attitude of general acceptance toward depicting instances of sexual harassment within the hospital setting [5].

In these shows, there are varying types of sexual harassment depicted; however, the most common type is telling sexual anecdotes or jokes. This displays a pattern of sexual harassment within the normal conversation between characters. In hospital environments, the use of this sort of language is not tolerated, as evidenced by the focus placed on sexual harassment in employee training as well as in medical and nursing student training. Unfortunately, this type of behavior is continually depicted on these shows. Even when these instances are featured as small anecdotes or jokes, this form of verbal harassment only serves to illustrate the visible surface of a deeper issue in the available media, and that this type of behavior is commonplace and/or widely tolerated.

This trend of verbal harassment is not only demonstrated through the telling of sexual anecdotes or jokes but also with making sexual comments about other individuals. This is the second most common type of harassment that we found depicted in our analysis. These findings are consistent with reports from Polce-Lynch et al. (2001), suggesting that TV shows based on workplace settings besides the medical field also depict people talking about sex as much as any other topic [6]. This raises concern as medical settings are highly volatile and, despite its inappropriate nature in other workplace settings, it can have an effect not only on the recipient of the harassment but also on the patients they are caring for. The frequency of sexual topics being brought up and used as a means of harassment lends credence to the idea that it is accepted as a norm.

Norms such as these are quite dangerous since, as previously mentioned, hospitals house a unique atmosphere where the process of work involves not one person but an entire care team. The team component is critical to the enhanced seriousness because multiple people have the opportunity to either stop the harassment or perpetuate it. Wong et al. (2019) examined trends in four medical TV shows with regard to sexual harassment and found that in many shows, including Scrubs, the effect of sexual harassment was perpetuated and even masked under the guise of comedic relief [2]. This research differs from our data, most significantly in the number of shows selected for analysis (four versus eight), and that the data analysis was conducted using a coding algorithm to record sexual harassment instances in the data entry tool.

The most pressing issue with the perpetuation of these instances of sexual harassment is that they serve to belittle members of a care team. Despite what is shown in the script, when these situations are applied to reality, they may affect both the team dynamic and the quality of care a patient receives. The darker side of harassment in these circumstances manifests in the clinical practice of the character. As the team members are more stressed and upset from harassment, there will often be a decline in the quality of their care. This can lead to a patient being hurt and the recipient of harassment being hurt as well [7].

The bridge from these shows to reality is such that there needs to be a fine line of distinction between the two. The unfortunate fact related to TV and media is that perception often becomes reality for many people. For those that watch TV and are influenced to go into medicine, they may not only absorb the aspects of the career but also absorb the environment surrounding it [8]. If that environment serves to perpetuate sexual harassment and harm to other members of the team, then there will be a likelihood that the viewer will interpret that as the prevalence of a culture of acceptance or laxity [9]. This laxity then translates into their perception when it comes to medical education and interactions in the medical units.

We have identified several limitations in our study. Firstly, the data gathered from our sample of eight medical TV shows may not be generalizable to all medical TV shows aired over the past few decades. Secondly, we did not abstract some data that were relevant to the description of sexual harassment instances, such as the race of the character performing the sexual harassment and the race of the victim of the sexual harassment, the power differential between attending physicians involved in sexual harassment, and the specific location where the sexual harassment occurred.

## Conclusions

Based on our analysis of the sample of medical TV shows, the victim of sexual harassment is often a female and an intern, and the character role often performing the sexual harassment act is an attending physician; the most common relationship pertaining to the act of sexual harassment is an attending physician toward an attending physician, the most common type of sexual harassment is “telling sexual anecdotes or jokes”, and the most frequent time period that sexual harassment was depicted was 2001-2005. In the current era, when we are collectively and publicly sensitive to the issue of sexual harassment and maintain a zero-tolerance attitude toward those who commit it, it is vital that we recognize where and when they can manifest. Avenues of culture and entertainment, such as media that portray the field of medicine, are important domains from where people can gain an understanding of how colleagues treat one another. The idea of taking notice of inappropriate/untoward remarks and actions, as well as correcting them both in the media and in medical education, is crucial to creating a mindful and socially aware clinical workforce. It ensures the safety of staff, patients, and the integrity of the field as a whole.
